# Lipase Immobilization on Silica Xerogel Treated with Protic Ionic Liquid and its Application in Biodiesel Production from Different Oils

**DOI:** 10.3390/ijms19071829

**Published:** 2018-06-21

**Authors:** Nayára B. Carvalho, Bruna T. Vidal, Anderson S. Barbosa, Matheus M. Pereira, Silvana Mattedi, Lisiane dos S. Freitas, Álvaro S. Lima, Cleide M. F. Soares

**Affiliations:** 1 Institute of Technology and Research, Avenida Murilo Dantas 300, Aracaju 49032-490, Sergipe, Brazil; nayara.eng@hotmail.com (N.B.C.); brunabtv@hotmail.com (B.T.V.); andinhobarbosa@hotmail.com (A.S.B.); matheus.pereira@ua.pt (M.M.P.); aslima2001@yahoo.com.br (A.S.L.); 2 Postgraduate in Process Engineering, Tiradentes University, Avenida Murilo Dantas 300, Aracaju 49032-490, Sergipe, Brazil; 3 Department of Chemical Engineering, Federal University of Bahia, Rua Prof. Aristides Novis, 02, Federação, Salvador 40210-630, Bahia, Brazil; silvana@ufba.br; 4 Department of Chemistry, Federal University of Sergipe, Av. Marechal Rondon, s/n, Jd. Rosa Elze, São Cristóvão 49100-000, Sergipe, Brazil; lisiane_santos_freitas@yahoo.com.br

**Keywords:** treated silica xerogel support, protic ionic liquid, lipase, immobilization, hydrolysis, transesterification

## Abstract

Treated silica xerogel with protic ionic liquid (PIL) and bifunctional agents (glutaraldehyde and epichlorohydrin) is a novel support strategy used in the effective immobilization of lipase from *Burkholderia cepacia* (LBC) by covalent binding. As biocatalysts with the highest activity recovery yields, LBC immobilized by covalent binding with epichlorohydrin without (203%) and with PIL (250%), was assessed by the following the hydrolysis reaction of olive oil and characterized biochemically (Michaelis–Menten constant, optimum pH and temperature, and operational stability). Further, the potential transesterification activity for three substrates: sunflower, soybean, and colza oils, was also determined, achieving a conversion of ethyl esters between 70 and 98%. The supports and the immobilized lipase systems were characterized using Fourier transform infrared spectra (FTIR), scanning electron microscopy (SEM), elemental analysis, and thermogravimetric (TG) analysis.

## 1. Introduction

Free or immobilized lipases are the most studied class of enzymes in biotechnology, receiving attention both in academia and in industry because of the recent improvement in biocatalytic enzyme performance [1,2,3]. *Burkholderia cepacia* lipase (LBC) is of microbial origin and is widely used in several biotransformations due to its high selectivity and stability and tolerance to solvents frequently used in the reaction medium [4]. Transesterification using LBC has been reported by several studies and with different oils: soybean [5], palm [6], cotton seed [7], jatropha [8], sunflowers [9], and colza [10].

Efficient immobilization is the result of the perfect matching of several factors depending on the enzyme, the process, the immobilization support, and the additives used for modification supports [11,12,13,14]. Therefore, the use of an appropriate support can influence the biocatalyst physical-chemical properties, such as diffusion and catalytic efficiency on each specific reaction system [15,16]. Thus, tailored solutions must be developed for each specific process of interest. Indeed, direct investigation of what occurs concerning the catalytic efficiency, morphology and physical chemistry, is quite a formidable task, and the differences observed in the performance of the immobilized enzyme system must be interpreted very carefully. In any study dealing with enzyme immobilization, the prerequisite is rigorous planning and reporting of experiments [17].

An alternative method for support enhancement in enzymatic immobilization is the modification of a silica surface support using an ionic liquid as an additive. The application of an additive has as its main goals a positive influence in the gel structure modification, the addition of silanol groups on the surface, an increase in surface area and pore size and the enhancement of the protective hydration layer on the enzyme, which prevents enzyme denaturation in the presence of alcohol [12,13,15,18,19].

Protic ionic liquids (PIL) are the result of the combination of an acid and a Brønsted base presenting high proton mobility, low cost, easy synthesis, and low toxicity, and favoring biocompatibility with lipases [12,13,20]. From the studies conducted by the present research group, of which this article forms part, it was found that the surface modification of silica with PIL for lipase immobilization by encapsulation positively influences the catalytic efficiency of the different lipases tested [13,21]. The literature suggests that surface modification of the support with aprotic IL positively influences the silica surface support due to the structure of the IL. However, up to now the surface modification of silica xerogel with protic ionic liquid for lipase from *Burkholderia cepacia* immobilized by physical adsorption or covalent binding has not been reported.

Surface modification of the silica support also occurs via the addition of specific functional groups and is a prerequisite for the immobilization of an enzyme by covalent binding to a solid surface [11,17]. Other surface modifications using e.g., silanized agents based on silanizing with organosilanes, occur by reaction between the reactive groups of alkoxy (methoxy, ethoxy) alkoxysilanes present in mono-, bi-, or tri-functional groups and the silanol on the support surface [22,23]. In addition, bifunctional agents or spacer arms (epichlorohydrin, glutaraldehyde, glyoxal, formaldehyde, carbodiimide, ethylenediamine, glycidol, carbonyldiimidazole, and others) are also used for the surface modification of silica, their function being to promote a strong attachment between the support (silanized or otherwise) and the immobilized enzyme. Bifunctional agents may interact with the groups present on the enzyme, such as hydroxyl, mercapto or amine groups, allowing a larger conformation and flexibility for the immobilized system [24,25].

In this context, the aim of the present work was the immobilization of lipase from *Burkholderia cepacia* by covalent binding onto supports obtained by a sol-gel technique treated with protic ionic liquid (PIL), N-methylmonoethanolamine pentanoate, and bifunctional agents (epichlorohydrin and glutaraldehyde). Moreover, evaluation of this potentially useful biocatalyst in hydrolysis and transesterification reactions was performed. In addition, biochemical characterization of the immobilized system under standard reaction conditions was performed (Michaelis–Menten constant, pH and temperature optimum and operational stability in the hydrolysis of olive oil). The supports and the immobilized system were characterized regarding particle size distribution, specific surface area, pore size distribution by Brunauer–Emmett–Teller (B.E.T. method), thermogravimetric analysis (TG), scanning electron microscopy (SEM), and Fourier transform infrared spectroscopy (FTIR) seeking to confirm the positive effect of silica xerogel surface modification by PIL on lipase immobilization.

## 2. Results and Discussion

### 2.1. Lipase Immobilization

The activity recovery yield of the two preparations of immobilized lipase by covalent binding onto silica xerogel supports obtained by a sol-gel technique treated (SIL) or no (SC) with ionic liquid and epichlorohydrin (CBE) or glutaraldehyde (CBG) was investigated using an olive oil hydrolysis reaction as a model [13,26,27,28]. The activity recovery yields carried out using CBG-SC, CBG-SIL, CBE-SC, and CBE-SIL were 21, 23, 203, and 250%, respectively. Between the two types of immobilization procedure for covalent binding, using glutaraldehyde or epichlorohydrin. Epichlorohydrin as the bifunctional agent showed higher activity recovery yield values. Therefore, the modification of the silica xerogel for immobilization showed improve of the 250% when compared to the other biocatalysts immobilized in this study, increasing up to 12 times.

According to Forde et al. [29] glutaraldehyde is frequently used for immobilization of soluble enzymes in order to increase the hydrophobicity of support, and authors report that lipases undergo hyperactivation in the presence of highly hydrophobic supports [30,31]. However, the positive influence of this bifunctional agent depends on the type of enzyme and support and according to the report by Minovska et al. [32], and although hyperactivation of lipase on glutaraldehyde-support is expected, a strong decrease on the activity was observed.

The low yield with conformational change in the enzyme during the stage of attachment to the support activated with glutaraldehyde could be due to possible functional agent toxicity to some enzymes [33,34]. On the other hand, hyperactivation of the enzyme was reached by activation with epichlorohydrin. These results could be attributed to different orientations of the enzyme on the support surface [35,36,37]. This behavior was also observed by Mendes et al. [38], in the immobilization of *Thermomyces lanuginosa* lipase by covalent binding on different supports (poly-hydroxybutyrate, bagasse and hybrid hydrogels). Immobilized lipases prepared with bifunctional agents such as glycidol and epichlorohydrin were more active in organic and aqueous media than those biocatalysts immobilized onto supports activated with glutaraldehyde agent.

The behavior observed by Mendes et al. [38] and Paula et al. [34] was further explained by Barbosa et al. [39], who reported that the optimal activation (in terms of reactivity) for silica supports is activation with two glutaraldehyde molecules per amino group on the support, generating a quite reactive (although not fully determined) structure with the amino residues of the enzyme. Moreover, glutaraldehyde dimers confer some hydrophobicity to the support surface, and they may affect the immobilization of some enzymes. For example, lipases exhibit so-called “interfacial activation” and this involves the movement of a polypeptide chain “lid” that may isolate the active site from the medium. This flexibility of the active site of lipases allows their functional properties (activity, selectivity, specificity) to be easily modulated, positively or negatively, through several techniques [40,41].

Based on these results, the active site of lipases immobilized on the support activated with epichlorohydrin was oriented toward reaction medium, which allowed access to the substrate. This difference may have been caused due to its high reactivity by the conformational modification of the enzyme during the fixation step on the glutaraldehyde-activated support, in which it alters the three-dimensional structure of the active site of the enzyme, rendering parts of the enzyme molecule inaccessible to the substrate [42].

Besides, when epichlorohydrin is used as a bifunctional agent, epoxy groups are formed on the support surface and only one hydroxyl group is needed for the reaction, compared to the two groups required by glutaraldehyde. This means that a higher number of active sites for enzyme binding are available on the silica xerogel activated with epichlorohydrin. This fact, as well as the known higher reactivity of epoxy groups, could explain the higher activity recovery yield achieved with epichlorohydrin as activating agent [43,44,45].

Biochemical, morphological and physicochemical characterization was performed only for the biocatalysts showing higher yields of immobilization. Thus, the characterization of lipase from *Burkholderia cepacia* immobilized by covalent binding with epichlorohydrin onto silica xerogel support without (SC) and with (SIL) ionic liquid is presented.

### 2.2. Biochemical Characterization

#### 2.2.1. Temperature and pH Effect on Lipase Activity

**Figure 1:**
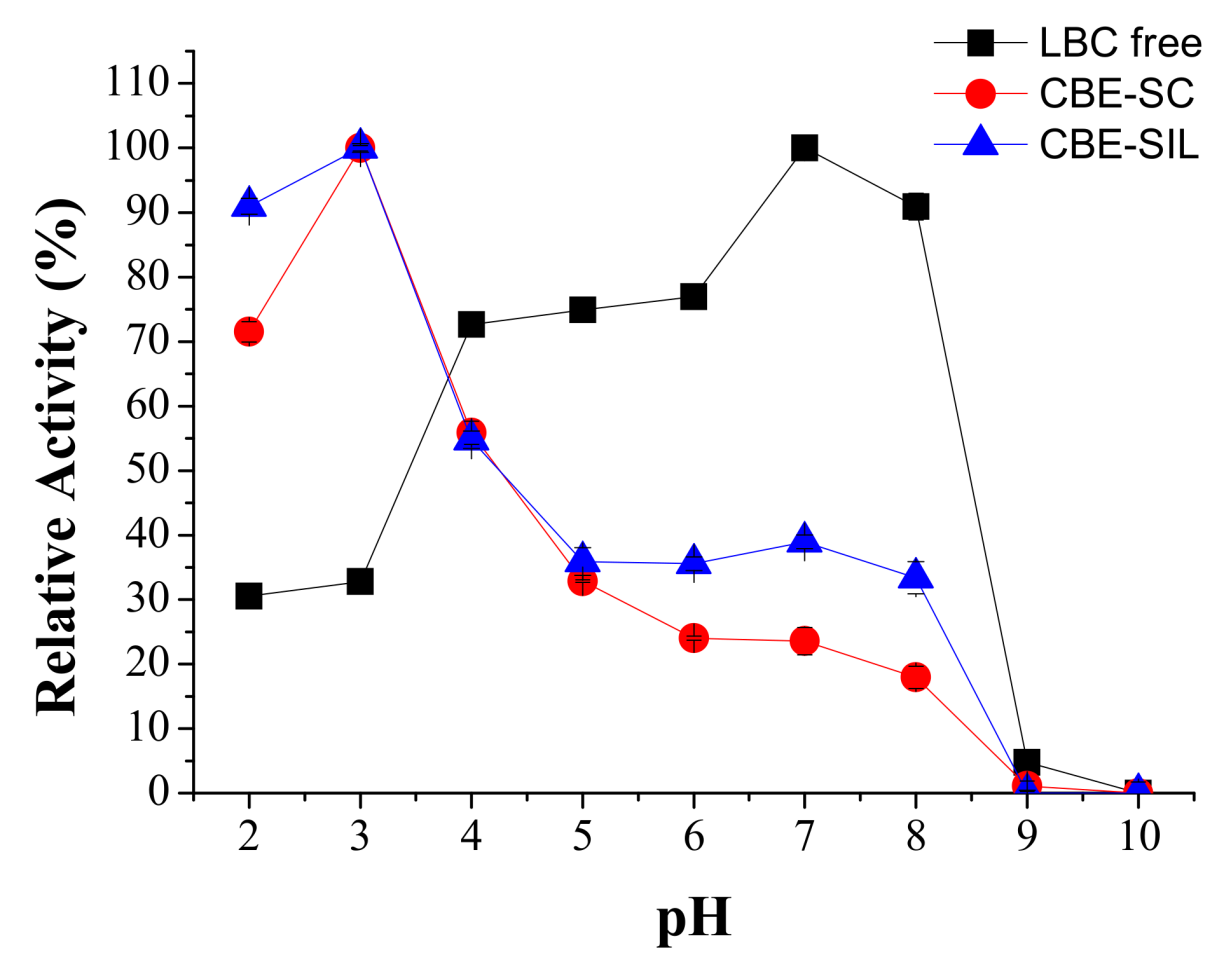
Effects on relative activity under various pH and temperature values. Ranging from pH 2 to pH 10 at 37 °C for lipase from *Burkholderia cepacia* free (LBC) (■), lipase immobilized by covalent binding with epichlorohydrin onto support produced without ionic liquid (CBE-SC) (●), lipase immobilized by covalent binding with epichlorohydrin onto support produced with IL (CBE-SIL) (▲).

In this study, pH and temperature effect were evaluated as well as their influence on the interaction of free lipase or immobilized lipase system with the surrounding microenvironment. For each immobilized system it was necessary to define the optimal pH since pH has a variety of effects on the ionization state, enzyme dissociation, and enzyme conformational change, and has a profound effect on hydrolytic activity after the immobilization process [46,47,48]. Figure 1 depicts the pH influence on relative activity for free lipase and immobilized.

For lipase from free *Burkholderia cepacia*, the optimal pH was 7.0, while the optimum pH for immobilized enzyme was shifted to 3.0. The change might be caused by the characteristics of the treated support and immobilization method (covalent binding) and can be attributed to alteration of various intermolecular interactions such as ion-dipole, dipolar, dispersion, and H-binding between the enzyme and the treated silica support with IL [18,48].

According to Hu et al. [16] immobilized lipase from *Burkholderia cepacia* in mesoporous silica SBA-15 modified by imidazole based ionic liquids was more stable to pH change from pH 7.0 pH 6.0–8.0, compared with that immobilized lipase in unmodified SBA-15. As indicated above, pH adaptability is associated with the charge of the supports. The presence of cations in ILs might reduce their sensitivity at low pH and improve the structure and the adaptability of the immobilized enzyme system. Thus the shift of optimal pH in this study was from neutral towards acid.

**Figure 2:**
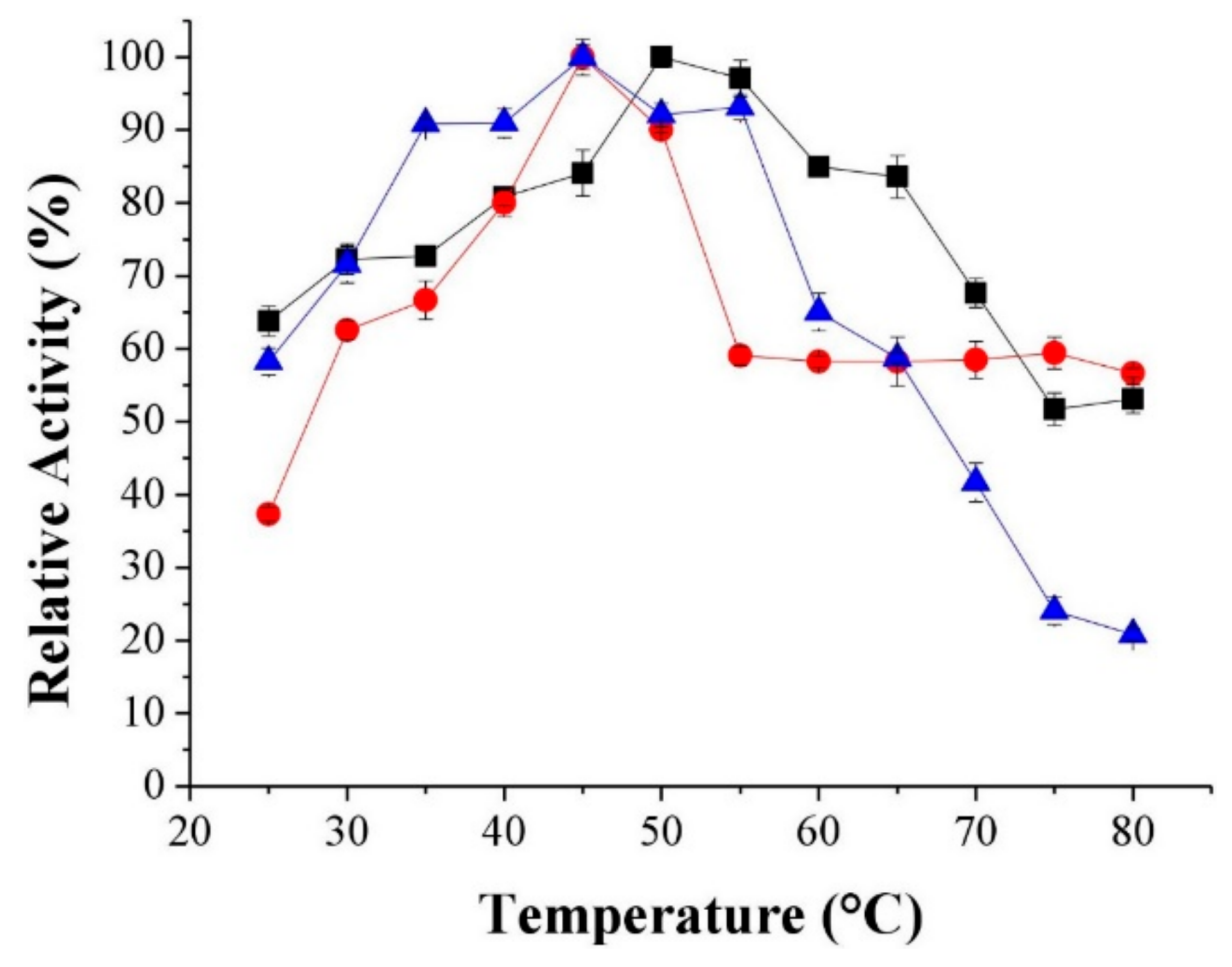
Effects on relative activity under ranging from 25 to 80 °C for lipase from *Burkholderia cepacia* free (LBC), pH 7.0 (■), lipase immobilized by covalent binding with epichlorohydrin onto support produced without IL (CBE-SC), pH 3.0 (●), lipase immobilized by covalent binding with epichlorohydrin onto support produced with IL a(CBE-SIL), pH 3.0 (▲).

The temperature dependence of the relative activity of free and immobilized system was investigated by measuring the hydrolysis of emulsified olive oil at different temperatures (from 25 °C up to 80 °C) and the results are shown in Figure 2. The optimal temperature for the free enzyme was approximately 50 °C, while for the immobilized enzymes the optimal temperature was 45 °C. This result shows that immobilization resulted in a lower activation energy for the enzyme. The higher activation energy for the free enzyme implies an increased energy barrier, meaning that the immobilized enzyme does not need an elevated temperature to attain its highest activity [48]. Similar results have been reported in the literature, where the optimum temperature of the immobilized lipase was in the range of 40–60 °C [12,20,49].

#### 2.2.2. Determination of Kinetic Parameters

The kinetic studies of lipase-catalyzed conversions have been much studied and more research is always needed because each process involves different parameters such as lipase type, lipase immobilization, the solvent used, temperature, reactant concentrations, and mass transfer limitations, as well as the parameters affecting the ping-pong bi-bi mechanism proposed for lipase [50,51].

Kinetic constants of free and immobilized LBC were determined by using olive oil emulsification solution as substrate in the hydrolysis reaction. The Michaelis–Menten constant *K_m_* and the initial maximum reaction velocity *V*_max_ of the immobilized LBC were calculated by nonlinear fitting using the program Origin^®^ 8.0., where *K_m_* determines the affinity of the enzyme for a particular substrate and *V*_max_ determines the maximum rate of reaction. Table 1, shows that the *K_m_* and *V*_max_ of the free enzyme were 249 mM and 1836 U·g^−1^, respectively. After immobilization, the biocatalysts had a higher *V*_max_ when compared to the free enzyme, 2718 and 3801 U·g^−1^ for CBE-SC and CBE-SIL respectively.

The apparent *V*_max_ values were consistent with the *K_m_* values. The results suggested that the IL modification increased the affinity of the enzyme and substrate. The lower the *K_m_* value the higher is the affinity of the enzyme to the substrate. According to Cabrera-Padilla et al. [27], the higher affinity for the substrate obtained in the case of the immobilized enzyme could be explained by the hydrophobic microenvironment of the immobilized enzyme attracting the substrate which is also hydrophobic, resulting in the obtained values for *K_m_*. Similar observations were made by Carvalho et al. [28] while evaluating the *K_m_* of lipase extracted from *Bacillus* sp., ITP-001 in the hydrolysis reaction of olive oil, where they obtained *K_m_* values of 105 and 14 mM for free and immobilized enzyme, respectively.

When comparing the lipase system immobilized onto supports control or treated with IL, it could be observed that the *K_m_* was higher for the biocatalysts immobilized onto supports treated with IL. This feature is possibly due to the greater porosity of the treated support and immobilization of lipase inside pores, indicating that the affinity towards the substrate was reduced when compared to enzyme immobilized only on the surface of the control support [44]. The maximum reaction speed of the immobilized system increased after immobilization of the enzyme. This suggests that the enzyme may have undergone a positive conformational change during immobilization, which affected the reaction rate, and could have influenced the diffusional mass transfer of substrate to the immobilized enzyme [1,27,52].

#### 2.2.3. Operational Stability

**Figure 3:**
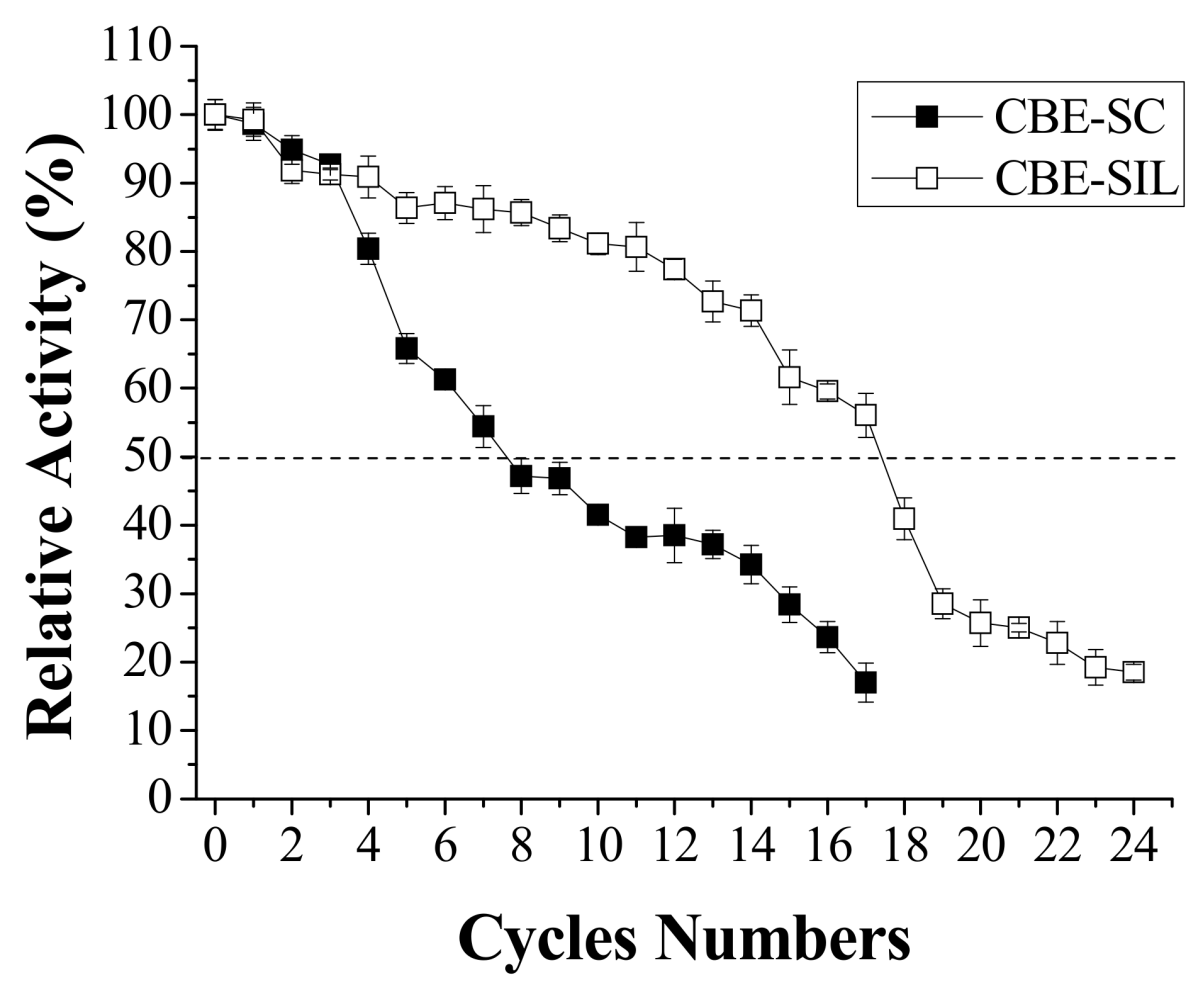
Operational stability of the lipase from *Burkholderia cepacia* immobilized by covalent binding. Reactional conditions were: 45 °C, pH 3.0, during 10 min and 80 rpm.

The operational stability of the immobilized enzyme system is one of the important characteristics that can help to reduce industrial process operation costs. This is because free enzymes are usually soluble and more unstable in the reaction medium, and it is necessary to constantly add new enzymatic charges and when enzymes are immobilized they have a higher stability and can be used in multiple cycles or in a continuous process. [48]. Figure 3 shows the variation of CBE-SC and CBE-SIL relative activity after reuse. Lower activities of CBE-SC were measured after four cycles of use, with a half-life of seven cycles and reaching 17% at 17 cycles. CBE-SIL maintained approximately 50% of its initial activity up the 17th cycle, which confirms that it has a more stable structure than CBE-SC. Similar results were found in works developed by Barbosa et al. [12] with LBC lipase immobilized by covalent binding on aerogel silica. The half-life of the biocatalyst immobilized onto modified support with IL increased from seven cycles to 10 cycles when compared to the immobilized biocatalyst under unmodified support.

Yang et al. [18], Hu et al. [16], and Zou et al. [53,54] showed greater variation of immobilized lipase by physical adsorption on functionalized mesoporous silica SBA-15 modified by ionic liquid, decreasing after each cycle and reducing rapidly in the initial period. This behavior was due possibly to the weak molecular interactions between the enzymes and supports through simple physical adsorption or it may also have been a conformational change in the enzyme structure during the successive batches. Therefore, other immobilization methods such as covalent binding should be considered to further improve the reusability of immobilized lipases. In addition, these studies demonstrate that besides the surface modification of silica xerogel, it is also necessary to choose an appropriate method of enzyme immobilization that provides stronger molecular interactions between enzymes and supports.

### 2.3. Morphological and Physicochemical Characterization

#### 2.3.1. Scanning Electron Microscopy (SEM)

**Figure 4:**
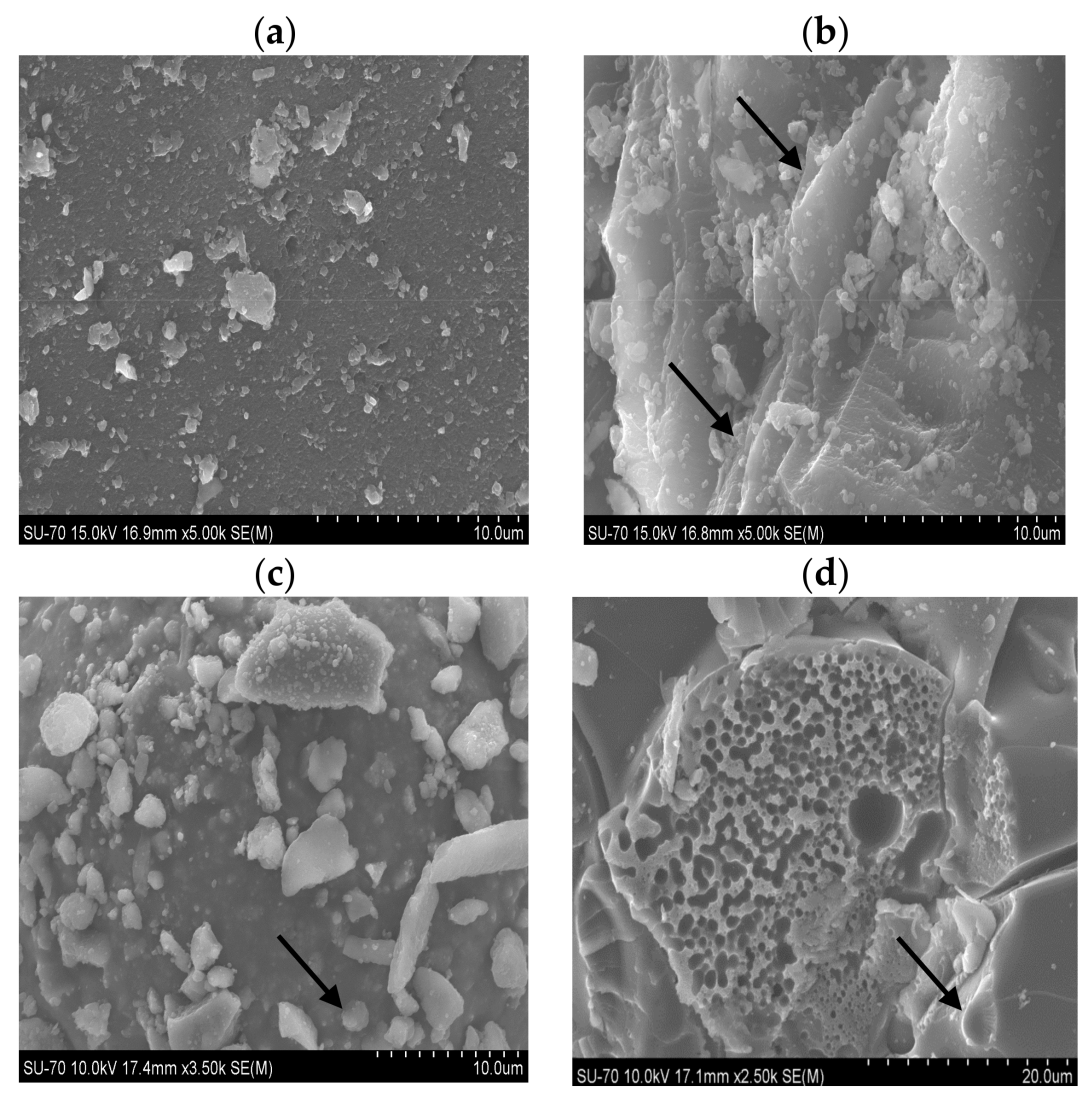
Scanning electron micrographs of SC (**a**); SIL indicating the black arrows a more porous and irregular surface (**b**) and CBE-SC (**c**) and CBE-SIL (**d**) indicating the black arrows possibly the presence of lipase due to its characteristic rounded shape.

SEM micrographs of the lipase from *Burkholderia cepacia* immobilized onto silica xerogel support prepared with ionic liquid and on the control support are shown in Figure 4. In Figure 4b, it can be observed that the support in the presence of IL showed high surface porosity, with formation of a layer of porous channels with irregular shapes and the support without IL and immobilized lipase presented low porosity (Figure 4a–c).

The micrographs in Figure 4d showed differences in the structure of the immobilized lipase system. Support produced using IL shows a predominance of pores for the immobilization of lipase by a covalent bifunctional agent such as epichlorohydrin. In conclusion, ionic liquid and the bifunctional agent promoted physical changes in the support and resulted in a more porous and efficient support for immobilization. According to Da Rós et al. [44], the SEM also allows the verification of morphological differences between pure and activated SiO_2_–Polyvinylalcohol supports. After the modification with epichlorohydrin, small fissures were verified, which may help the fixation of the enzyme on the support.

The higher activity recovery yield obtained indicated that some sort of change occurs in the mechanism of the enzyme system on treated silica xerogel with IL and epichlorohydrin. It was also shown that the activity of CBE-SIL was better than that of CBE-SC, which reinforces the advantages of IL use. Speculation about the mechanism is possible with data from biochemical and physicochemical characterization.

#### 2.3.2. Specific Surface Area and Porosity

The determination of the specific surface area and pore size is based on the volume of nitrogen gas adsorbed at various pressures at 77 K (−196 degrees Celsius). The results of surface area measurement, diameter and pore volume for the silica xerogel support and immobilized system are shown in Table 2.

The results for the specific surface area indicated a increase of specific surface area when the ionic liquid was added to the support synthesis (from 799.5 m²·g^−1^ (SC) to 853.5 m²·g^−1^ (SIL). This behavior was also reported in studies using supports with IL and enzyme by sol-gel encapsulation by Souza et al. [13] and Barbosa et al. [21]. The surface area of CBE-SIL was always larger than CBE-SC at the same loading, which would be an advantage for enzyme dispersion and substrate diffusion.

In studies carried out by Yang et al. [18] mesoporous silica SBA-15 was modified by carboxyl-functionalized ionic liquid. The prepared support was used to immobilize porcine pancreatic lipase by physical adsorption and covalent binding. After modification, the pore size, pore volume, and surface area of SBA-15 decreased. The drop in these parameters could be attributed to changes of the mesopore domains and the occupation of the functional groups in the mesopore channels [12,53,55].

After treatment of the control support without ionic liquid (SC) with epichlorohydrin, the surface area decreased by 50% and the pore volume increased. The increased volume of the pores is possibly due to the formation of rigid spacer arms and the formation of porous channels. Similar results were observed for the surface area of silica xerogel supported with IL (CBE-SIL), but the pore volume decreased, since the spacer arms for the immobilization of lipase were retained not only at the surface but also in the interstices of the support, preventing the adsorption of nitrogen and consequently increasing the diameter and pore volume [56,57].

The results showed an increase of pores provided by the ionic liquid, generating greater lipase load in the interstices, and maintaining the water retaining layer of enzyme hydration. The best activity recovery yield, ester conversion and improved operational stability were also confirmed. Poppe et al. [58] also describe the stability of lipases from *Rhizomucor miehei* and *Candida antarctica* type B on modified Immobead 150 supports, showing the positive effect of immobilization on the thermal stability of lipase enzymes.

#### 2.3.3. Thermogravimetric Analysis

The mass loss observed from TG curves in the range 25–1000 °C allowed the observation of enzyme degradation, water extraction from the surface and the pores, definitive carbonization, and others factor associated with condensation of silanol groups. The thermogravimetric curves for the samples (LBC, SC, SIL, CBE-SC or CBE-SIL) were divided into three regions as a function of temperature: I (25–200 °C), II (220–600 °C) and III (600–1000 °C) (Table 3).

Table 3 shows that in the area between II and III LBC underwent approximately 100% mass loss, probably due to decomposition of the enzyme organic compounds [21,43]. In Poppe et al. [58], water loss from free lipase in the same temperature range was also seen, and it was concluded that temperatures higher than 200 °C are required to remove tightly bound water from lipase. SC mass loss was 21% in area I and 5% in area II, different to SIL which lost 20% in area I and 10% in area II. Both lost about 1.5% in region III.

The weight loss in area I can be attributed to the presence of unreacted silanol groups from the tetraethoxysilane (TEOS), which are present in the silica due to incomplete sol-gel reactions. The difference in mass loss of the mass media in area II can be attributed to the presence of water in the pores, since the ionic liquid is hydrophobic and can retain water during the process of support synthesis. Additional mass loss can also be associated with the condensation of silanol groups and some loss of organic constituents (C, H, O and N) in the form of volatiles either present or formed at the beginning of organic decomposition, including decomposition of lipase [13,21].

The immobilized lipase system weight loss was more pronounced in region I, at 23%, which could be associated with the desorption of water molecules from the support and the decomposition of organic and amino groups from the enzyme present on the surface [43]. For the immobilized lipase system onto support synthesized with ionic liquid, CBE-SIL, the highest weight loss was observed in region II, at 15%, due to the presence of enzyme on the surface and within the porous support.

The results show an increase in pores provided by the ionic liquid, generating a higher lipase load in deeper pores inside the support, besides promoting the retention of the water layer responsible for the hydration of the enzyme. This behavior may be justified by the lower mass loss for the biocatalysts immobilized CBE-SIL and thereby improving thermal stability. The best yield of immobilization, ester conversion and operational stability were also confirmed. Poppe et al. [58] also describe the improved stability of *Rhizomucor miehei* and *Candida antarctica* type B lipases in modified Immobead 150 supports.

#### 2.3.4. Fourier Transform Infrared Spectroscopy

**Figure 5:**
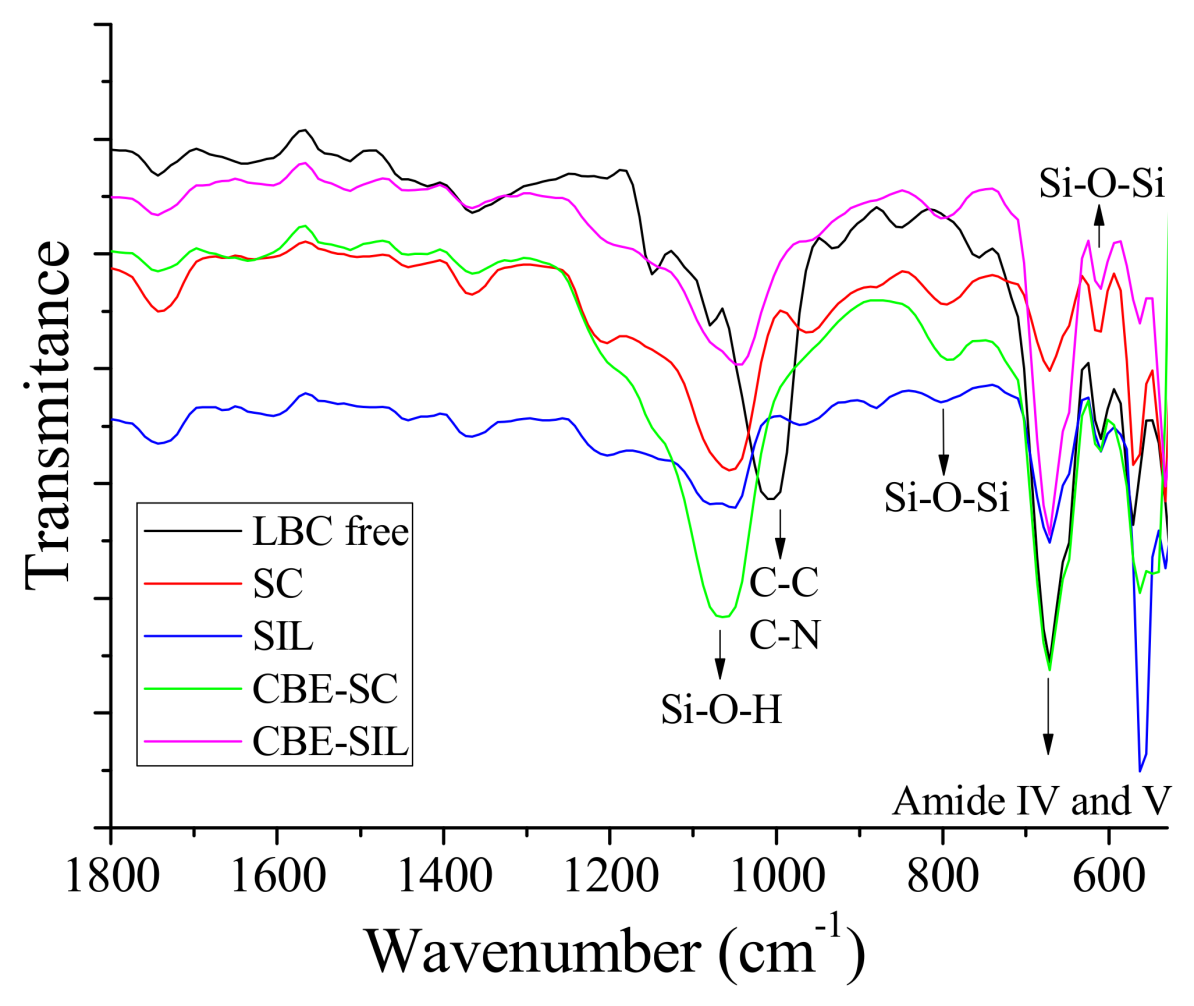
FTIR spectra of the lipase from *Burkholderia cepacia* (LBC free), control silica (SC), silica produced with protic ionic liquid (SIL), lipase immobilized by covalent binding with epichlorohydrin onto silica control (CBE-SC) or lipase immobilized by covalent binding with epichlorohydrin onto silica produced with protic ionic liquid (CBE-SIL).

**Figure 6:**
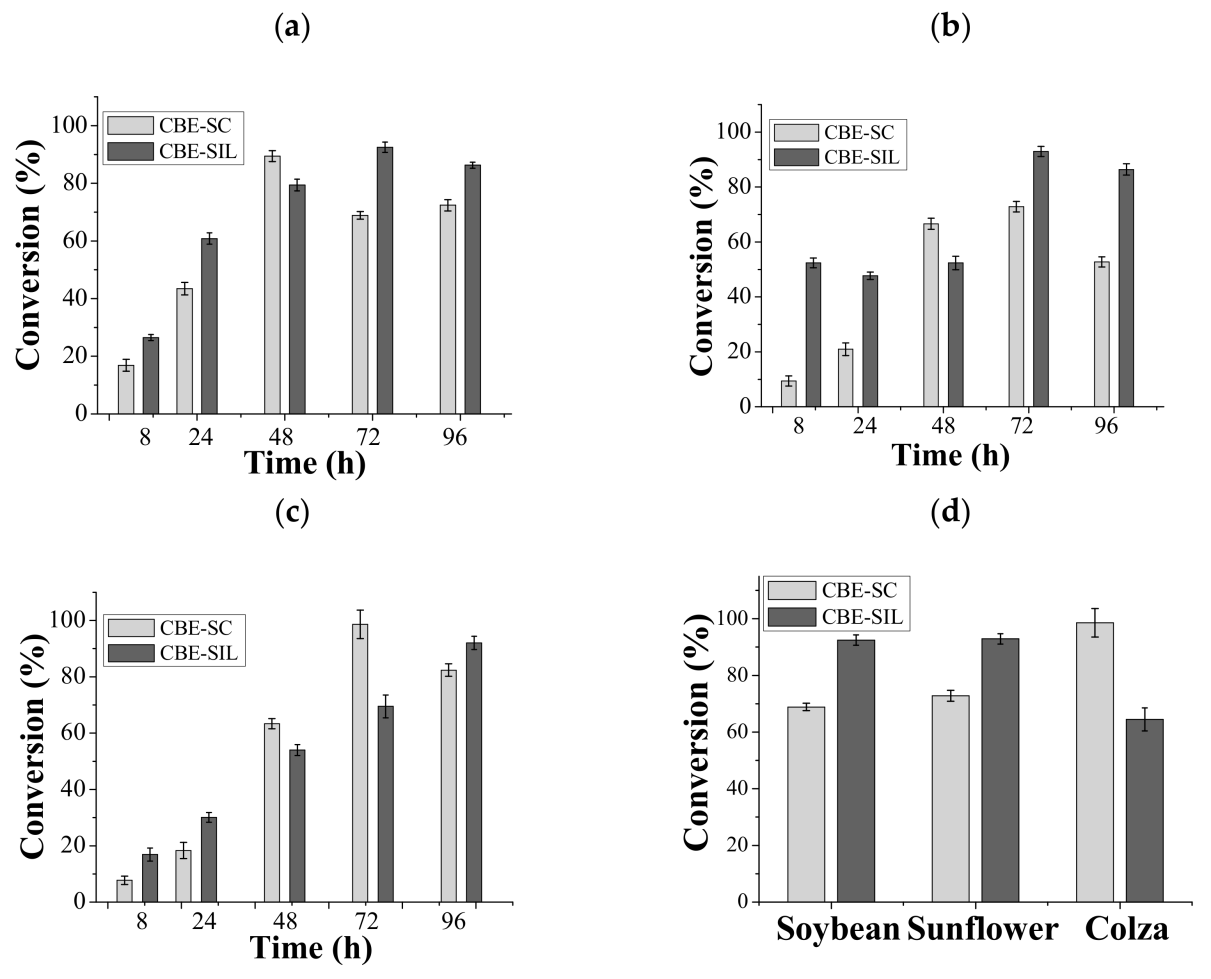
Time course of ethyl ester conversion using lipase from *Burkholderia cepacia* immobilized by covalent binding for different oils: soybean (**a**), sunflower (**b**) or colza (**c**). Experiments considered under a 40 °C, 96 h of reaction and stirring at 80 rpm. In image (**d**) a comparison between the three oil types for aliquots taken at 72 h of reaction under the already described conditions, is seen.

The efficiency of lipase immobilization on the silica xerogel support with additives was also assessed by Fourier transform infrared spectroscopy (Figure 5). Typical FTIR spectra of the silica xerogel support with or without IL, as well as of the lipase from *Burkholderia cepacia* free and their immobilized forms are presented in Figure 6 for comparison purposes.

Free enzyme displayed a typical protein spectrum with bands associated with their characteristic amides (IV and V group) in the region around 695 cm^−1^, as reported by Portaccio et al. [59] and Barbosa et al. [12]. When BC is immobilized onto SC or SIL there is of the peaks of the enzyme and silica support. The observed bands represent amides IV and V are also present in the spectra of CBE-SC and CBE-SIL, revealing the presence of LBC in the immobilized systems. Amide IV and V are, respectively, due to OCN bending and out-of-plane NH bending [59]. Although a detailed assignment is not possible at the present time because of the complexity involved, the observed changes in the vibrational spectra corroborate the existence of different binding modes of the enzyme to the support.

In addition, there is enhancement of the vibrational peaks around 1100–1000 cm^−1^ are associated with the C–C and C–N composite vibrations of the protein chain [12]. No additional bands in the spectrum of any of the immobilized systems have been observed, this suggests that the same bands found in the systems are of the same nature as the free lipase.

The FTIR spectra of SC, SIL, CBE-SC and CBE-SIL showed absorption bands at around 620 cm^−1^. These obvious peaks were characteristic vibrations of the mesoporous framework Si–O–Si [13,16,53]. Si–O–H peaks at 1100–900 cm^−1^ are also characteristic absorptions that indicate the formation of the bonds between silica molecules as a result of polymerization of TEOS during the formation of the silica [12,43,60]. The spectra of the native support materials confirmed that the functional groups of all support materials were accessible for the subsequent immobilization reactions.

### 2.4. Transesterification Activity

Figure 6 shows the conversion to ethyl esters by transesterification of three oil types using lipase immobilized by covalent binding onto silica xerogel without protic ionic liquid (CBE-SC) and with protic ionic liquid (CBE-SIL).

Three oil types (soybean, colza and sunflower oil) were used to assess transesterification performance of the immobilized lipases. Lipase immobilized by covalent binding onto silica xerogel without protic ionic liquid (CBE-SC) achieved 89% conversion of ethyl esters in 48 h for soybean oil. However, using the biocatalyst immobilized onto treated silica xerogel with protic ionic liquid (CBE-SIL) conversion of soybean oil achieved almost 93% (72 h) ethyl ester conversion. For colza, the CBE-SC was more efficient reaching 98% conversion in 72 h. For sunflower oil, a 92% conversion was obtained after 72 h when using the CBE-SIL and 73% using the CBE-SC after 72 h. Therefore, it can be considered that lipase from *Burkholderia cepacia* immobilized by covalent binding was effective for the transesterification reaction.

In the literature, there are no studies using lipases immobilized by covalent binding to treated silica xerogel with IL for the synthesis of ethyl esters. Oliveira et al. [26] performed the encapsulation of LBC with a protic ionic liquid (*N*-methylmonoethanolamine pentanoate) and applied it in the transesterification of babassu oil and ethanol (molar ratio 1:7). Conversions of 52% were obtained in conventional medium. Only after increasing the enzyme load through covalent binding in the already immobilized biocatalyst was it possible to see an increase in the reaction performance leading to a 98% conversion (96 h).

It can be concluded that using ionic liquid for silica xerogel surface modification for covalent binding immobilization promoted higher conversions, without or with ionic liquid using a conventional process, between 48 and 96 h. Figure 6d shows the highest conversions obtained at 72 h. It can be concluded that the immobilized system onto support produced using ionic liquid presented higher conversion in ethyl esters for soybean and sunflower oils. For colza oil a higher conversion percentage was obtained for the biocatalyst immobilized on control support and different from the other oils showed a slow and gradual increase in conversion to ethyl esters. This different profile found for colza oil may be associated with physical-chemical factors, such as viscosity. Colza oil is more viscous when compared to other soybean and sunflower oils that have similar viscosity. The higher viscosity value may have made it difficult to increase the mass transfer rate due to the need for more time to be able to penetrate the support and reach the active site of the enzyme inside the pores.

Therefore, the use of immobilized biocatalysts in the production of ethyl ester is an interesting alternative that can be applied in reactions under moderate temperatures, as well as free lipase, but also in a slightly higher temperature range as proven in thermal stability thus increasing the speed in conversion rates. Also, unlike chemical catalysis which works better with methanol, immobilized enzymes seem to prefer ethanol [61,62,63]. Many vegetable oils can be used, such as soybean, sunflower, palm, colza, rapeseed, castor oil, cottonseed, jatropha, rice bran, coconut, hazelnut, pistachio, karanja, neam, mahu, caster, safallow, and jojoba [61,64,65,66].

The main differences among these vegetable oils are the fatty acid compositions, which strongly affect the production of esters. According to Resolution No. 482 (Technical Rules for Identity Fixation and Quality of Vegetable Fats and Oils), of 23 September 1999 ANVISA (National Health Surveillance Agency) from Brazil [67] the vegetable oils used in this study (sunflower soybean and colza) are compounds mostly containing fatty acids of between 16 and 18 carbons, as shown in Table 4.

According to Polaina et al. [68] substances such as phospholipids and waxes in oils may negatively affect enzyme activity. The observed differences in transesterification by the immobilized lipase are possibly due to the fatty acid composition of the oils studied (Table 4). The highest conversion was obtained for colza oil (98%). Although he did not specifically study the formation of oleate and linoleate ethyl, assessing the fatty acid content of the oils used, the highest conversion was possibly due to the greater presence of oleic and linoleic acid present in oils in study when compared to the others acids.

Da Rós et al. [44] concluded that there was no significant difference in transesterification of babassu oil and tallow beef when using lipase from *Burkholderia cepacia* immobilized by covalent binding on two different non-commercial matrices, an inorganic matrix (niobium oxide, Nb_2_O_5_) and a hybrid matrix (polysiloxane–polyvinyl alcohol, SiO_2_–PVA). Independent of the feedstock, both immobilized derivatives were able to form the main fatty acids ester from these raw materials. However, both reaction rates and yields were influenced by the support and activating agents used for immobilizing the lipase. This same effect can be observed when used lipase from *Burkholderia cepacia* immobilized onto supports produced with or without ionic liquid in this study.

## 3. Material and Methods

### 3.1. Material

Lipase from *Burkholderia cepacia* (Amano Lipase, ≥ 2200 U·g^−1^, pH 7.0, 50 °C) was purchased from Sigma Aldrich (Tokyo, Japan). The silane precursor tetraethoxysilane (TEOS) was supplied by Across Organic (Morris, NJ, USA) and used without further purification. Ethanol (99% pure), ammonia (28% pure), hydrochloric acid (36% pure) and arabic gum were obtained from Synth (São Paulo, Brazil). Water was purified by reverse osmosis and deionized through a Milli-Q four-cartridge organic-free water purification system. Olive (low acidity), sunflower, soybean, and colza oils were purchased at a local market. For covalent binding the bifunctional agents glutaraldehyde or epichlorohydrin from Sigma Aldrich were used. The protic ionic liquid N-methylmonoethanolamine pentanoate was supplied by the Federal University of Bahia (UFBA). Other chemicals were of analytical grade and used as purchased.

### 3.2. Xerogel Silica Preparation

The methodology used was as previously established by Souza et al. [13] with some modifications. For the mesoporous xerogel silica treated with ionic liquid (SIL), 1% (*w*/*v*) of *N*-methylmonoethanolamine pentanoate was added during the synthesis of the support. For the silica control (SC) this step was excluded. Two supports, the xerogel silica control support produced without ionic liquid and a xerogel silica support treated using ionic liquid, were obtained for functionalization with bifunctional agents and subsequent immobilization.

### 3.3. Lipase Immobilization

**Scheme 1:**
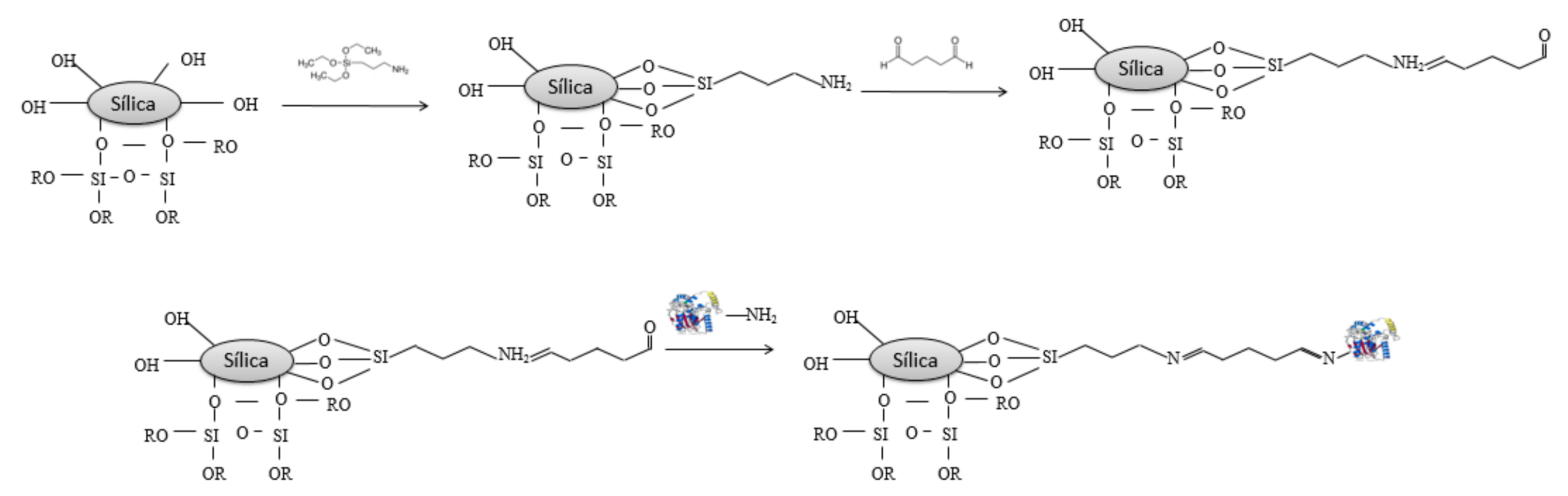
Possible reaction mechanisms of the silica support with functional activating agents glutaraldehyde and lipase.

Lipase from *Burkholderia cepacia* (LBC) was immobilized by covalent binding on the xerogel silica support (SC or SIL) using two bifunctional agents: glutaraldehyde or epichlorohydrin. For covalent binding with glutaraldehyde the xerogel silica support two steps were performed: in the first, the supports was silanized with γ-APTS (γ-aminopropyltriethoxysilane) and in the second followed by a reaction with glutaraldehyde solution. Soon after the functionalization of the supports, the lipase was placed in contact with the support and immobilized according to the procedure described by Soares et al. [43] with reported modifications (Scheme 1).

The mesoporous xerogel silica (SC or SIL) (1 g dry wt) was soaked previously in hexane under agitation (100 rpm) for 15 min. A 0.25 g lipase aliquot was dissolved in 10 mL of distilled water and then added to the solution. The fixation of lipase onto the support was performed under agitation for 3 h at room temperature and followed by an additional period of 18 h under static conditions at 4 °C. The derivative was filtered and thoroughly rinsed with hexane. All two preparations of immobilized biocatalysts onto support control (SC) or support treated with ionic liquid (SIL) of were denominated CBG-SC and CBG-SIL, respectively.

**Scheme 2:**
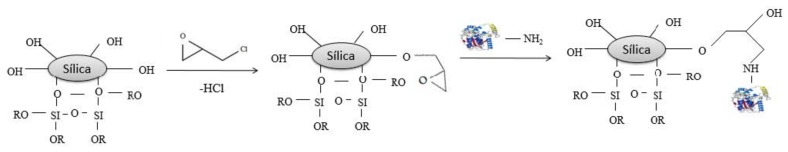
Possible mechanism reactions for silica support with functional activating agents epichlorohydrin and lipase.

For epichlorohydrin as bifunctional agent, lipase was immobilized following the methodology of Soares et al. [43], with slight modifications (Scheme 2). Functionalization of the xerogel silica was carried out with epichlorohydrin at 2.5 % (*w*/*v*) diluted in pH 7.5 in the ratio 1:10 (support:solution—*w*/*v*^1^) for 1 h at room temperature, followed by washings with distilled water. Support functionalized was soaked into hexane under stirring (100 rpm) for 1 h at 25 °C. Then, excess of hexane was removed and lipase was added at a ratio of 1:4 (g_enzyme_:g_support_), 0.25 g_lipase_/g_support_. PEG-1500 (5 mg·g^−1^) was added together with the enzyme solution at a fixed amount (100 µL·g^−1^ of support). Lipase-support system was maintained in contact for 16 h at 4 °C under static conditions. The immobilized systems onto silica control and treated with IL functionalized with epichlorohydrin were denominated CBE-SC and CBE-SIL, respectively.

### 3.4. Biochemical Characterization

#### 3.4.1. Enzymatic Hydrolysis

Enzymatic hydrolysis activities of free or immobilized lipase were assayed using the olive oil emulsion method according to literature [69]. The liberated fatty acids in enzymatic hydrolysis were titrated with potassium hydroxide solution (0.04 M) in the presence of phenolphthalein as indicator. All enzymatic activity determinations were replicated at least three times. One unit (U) of enzyme activity was defined as the amount of enzyme liberating 1 µmol of free fatty acid per min (µmol·min^−^¹). Analyses of hydrolytic activities were carried out on the lipase loading solution and the immobilized preparations to determine the yield immobilization, Y_a_ (%), according to Equation (1) [70]:

(1) $$Y_{a}(\%)=\frac{U_{s}}{U_{0}}\times 100$$

where: U_S_ corresponds to the total enzymatic activity immobilized on the support, and U_0_ represents the enzyme units offered for immobilization.

#### 3.4.2. Temperature and pH Effect on Lipase Activity in the Hydrolysis Reaction

The effect of pH on the activity of free and immobilized lipase was analyzed between pH 2.0–10.0 at 37 °C. The buffers used were 0.1 M citric acid-sodium citrate (pH 2.0–5.0), 0.1 M potassium phosphate (pH 5.0–8.0), and 0.1 M bicarbonate-carbonate (pH 8.0–10). The optimal temperature for activity of the immobilized lipase system was determined in the range of 25–80 °C, at the previously determined optimum pH.

#### 3.4.3. Kinetic Parameters in the Hydrolysis Reaction

To calculate the Michaelis–Menten constant (*K_m_* and *V_max_*), reaction systems were prepared containing fatty acids at concentrations ranging from 37 to 2232 mM obtained from emulsions containing different proportions of olive oil (10–90%) and aqueous solution of Arabic gum (7% *w*/*v*), in accordance with the proposed method by Cabrera-Padilla et al. [27]. The Michaelis–Menten constant (*K_m_*) and the concentration of substrate at which half of the maximum reaction rate (*V*_max_) was reached were calculated by nonlinear fitting using the program Origin 8.0.

#### 3.4.4. Operational Stability

The cycles of use of the immobilized lipase system was performed in hydrolysis reactions (olive oil) in consecutive batches. Each batch consisted of 10 min of hydrolysis reaction at the optimum reaction pH and temperature. At the end of each batch, the immobilized system was removed from the reaction medium, and washed with hexane in order to remove any substrate or product retained in the support. The immobilized system was then reused for another reaction cycle using new substrates. The relative activity of each run was calculated by setting the original activity as 100%.

### 3.5. Morphological and Physicochemical Characterization

After determining the highest yields for both preparations (CBE-SC and CBE-SIL), three control preparations (LBC, SC and SIL) were used for morphological and physicochemical characterization. The surface area of the xerogel silica support and the immobilized system were measured by adsorption, using nitrogen as the adsorbate according to Barbosa et al. [12]. The surface area (m^2^·g^−1^) was calculated using the Brunauer, Emmett, and Teller (B.E.T.) method. Pore volume (cm^3^·g^−1^), diameter (Å) and area distributions based on BJH calculation were evaluated by the B.E.T. apparatus software (Model NOVA—Surface Area & Pore Size Analyser, Quantschrome Instruments, Florida, USA). Thermogravimetric analyses (TG) curves were performed by TG, PerkinElmer Pyris1 apparatus, under a nitrogen atmosphere that started at room temperature and went up to 1000 °C, with a heating rate of 10 °C min^−1^.

The thermogram was divided into three regions as a function of temperature and the percentage of the mass loss was calculated using Equation (2).

(2) $$Mass\;Loss(\%)=\left(\frac{w_{f}-w_{i}}{w_{f}}\right)\times 100$$

in which *w_i_* and *w_f_* are the initial and final weight of samples, respectively.

Scanning electron microscopy (model Hitachi S4100i, accelerating potential of 30 kV to 500 V and 15 A resolution) was also used to characterize the micro-structural changes and the surface characteristics of the support with and without the treatment with IL and biocatalysts immobilized on the respective ones. The samples were fixed by means of double face tape for later analysis. The samples of LBC, SC, SIL, CBE-SC, and CBE-SIL were submitted to FTIR analysis (PerkinElmer). Spectra were obtained in the wavelength range 500 to 4000 cm^−1^.

### 3.6. Transesterification Reaction

The immobilized lipase systems with higher yields of immobilization were used for biochemical characterization in an olive oil hydrolysis and vegetable oil (soybean, colza, and sunflower) transesterification reaction. Transesterifications reactions catalyzed by lipase from *Burkholderia cepacia* were performed under the conditions proposed by Oliveira et al. [26] with some modifications. The reactions (5 g of total mass) were carried out in batch reactors, submerged in a thermostatic bath to keep each mixture at constant temperature and under agitation (80 rpm). The reaction was started by mixing oil (colza, soybean or sunflower oil) and ethyl alcohol (molar ratio 1:7), with 20% (*w*/*w*) of immobilized biocatalyst for up to 96 h at 40 °C.

The ethyl ester production were analyzed by gas chromatography (Shimadzu 2010, Shimadzu Corporation, Kyoto, Japan) equipped with FID (flame ionization detector) and CARBOWAX (30 m × 0.25 mm × 0.25 mm) column. The column temperature was kept at 140 °C for 1 min, heated to 180 °C at a rate of 4 °C/min, maintained for 2 min, then increased to 230 °C at a rate of 10 °C/min and kept constant for 10 min. The temperatures of the injector and detector were set to 250 °C. The injection volume was 1 µL. The internal standard was methyl heptadecanoate. Ethyl ester conversion (%) was defined as the observed amount of product divided by the theoretical yield if all the oil was converted.

The yield calculation in esters was carried out based on the mass and areas under the peaks corresponding to the ethyl esters, and on the internal standard, using Equation (3). Conversion analyses were performed in triplicate.

(3) $$Conversion(\%)=\frac{\left(A_{E}-A_{IS}\right)}{A_{IS}}\times \frac{\left(C_{IS}\right)}{C_{E}}\times 100$$

where: A_E_ = sum of the peak areas corresponding to the esters in the sample; A_IS_ = peak area corresponding to the internal standard; C_IS_ = internal standard concentration; C_E_ = esters concentration.

## 4. Conclusions

Surface treated silica supports were used to immobilize the lipase from *Burkholderia cepacia* by covalent binding. The addition of protic ionic liquid (PIL) during the synthesis of the xerogel silica support by a sol-gel method resulted in an improvement in their morphological and physicochemical characteristics. Bifunctional agents are an important factor in the yield of enzyme immobilization, and when epichlorohydrin was used, activity recovery yields of up to 250% for lipase immobilized onto treated xerogel silica by covalent binding were obtained. The kinetic parameters corroborate the results of the activity recovery yield. Therefore, careful selection of the support and the immobilization conditions and techniques is important for the generation of stable enzyme-support composite; improvement of the operational stability; achieving favorable temperature and pH optima and kinetic parameters; and the efficient conversion of esters, 98% for colza oil. Additionally, the advantage of ionic liquid treated SiO_2_ as support can depend on the reaction for which the immobilized enzyme is used.

## Figures and Tables

**Table 1:** The Michaelis constant (*K_m_*) and the maximum reaction velocity (*V*_max_) of the free enzyme determined by measuring initial rates of the reaction. Where free lipase from *Burkholderia cepacia* (LBC), immobilized by covalent binding without ionic liquid (CBE-SC) and immobilized with ionic liquid (CBE–SIL), are compared.

Biocatalysts	*V_max_* (U·g^−1^)	*K_m_* (mM)
LBC	1836 ± 141	249 ± 31
CBE-SC	2718 ± 237	99 ± 14
CBE-SIL	3801 ± 296	149 ± 25

**Table 2:** Physicochemical properties of silica xerogel supports (SC or SIL) and lipase from *Burkholderia cepacia* immobilized by covalent binding onto control silica (CBE-SC) or silica produced with protic ionic liquid (CBE-SIL).

Sample	Superficial Area (m^2^·g^−1^)	Pore Volume (cm^3^·g^−1^)	Pore Diameter (Å)
SC	799.5	0.57	30.25
SIL	853.5	1.02	41.76
CBE-SC	349.4	0.67	42.55
CBE-SIL	371.8	0.35	46.03

**Table 3:** Partial mass loss of lipase from *Burkholderia cepacia* (LBC), control silica xerogel (SC), silica xerogel produced with protic ionic liquid (SIL), lipase immobilized by covalent binding with epichlorohydrin on control silica (CBE-SC) or lipase immobilized by covalent binding with epichlorohydrin onto silica produced with protic ionic liquid (CBE-SIL).

Samples	Temperature (°C)	Partial Mass Loss (%)
LBC	25–200	8.0
	200–600	83.5
	600–1000	8.4
SC	25–200	21.2
	200–600	4.9
	600–1000	1.4
SIL	25–200	20.0
	200–600	9.8
	600–1000	1.5
CBE-SC	25–200	23.4
	200–600	17.7
	600–1000	2.2
CBE-SIL	25–200	10.2
	200–600	15.1
	600–1000	1.7

**Table 4:** Composition of soybean, sunflower and colza oil fatty acids, comparing fatty acids, carbon chain and their nomenclature.

Fatty Acids	Nomenclature	Soybean Oil	Sunflower Oil	Colza Oil
C 14:0	Myristic	<0.5	<0.5	<0.2
C 16:0	Palmitic	7.0–14.0	3.0–10.0	2.5–6.5
C 16:1	Palmitoleic	<0.5	<1.0	<0.6
C 18:0	Stearic	1.4–5.5	1.0–10.0	0.8–3.0
C 18:1	Oleic	19.0–30.0	14.0–35.0	53.0–70.0
C 18:2	Linoleic	44.0–62.0	55.0–75.0	15.0–30.0
C 18:3	Linolenic	4.0–11.0	<0.3	5.0–13.0
C 20:0	Arachidic	<1.0	<1.5	0.1–1.2
C 20:1	Eicosenoic	<1.0	<0.5	0.1–4.3
C 22:0	Behenic	<0.5	<1.0	<0.6
C 22:1	Erucic		<0.5	<2.0
C 24:0	Lignoceric		<0.5	<0.2
C 24:1	Tetracosenóico		<0.5	<0.2

From ANVISA (National Health Surveillance Agency) [67].

## Abbreviations

LBC	*Burkholderia cepacia* lipase
CB	Covalent binding
CBE-SC	LBC immobilized by covalent binding onto xerogel silica control and with epichlorohydrin
CBE-SIL	LBC immobilized by covalent binding onto xerogel silica treated and with epichlorohydrin
CBG-SC	LBC immobilized by covalent binding onto xerogel silica control and with glutaraldehyde
CBG-SIL	LBC immobilized by covalent binding onto xerogel silica treated and with glutaraldehyde
CG-FID	Gas chromatography–Flame Ionization Detector
FTIR	Fourier-transform infrared spectroscopy
IL	Ionic liquids
PIL	Protic ionic liquid
SC	Xerogel silica control
SEM	Scanning electron microscopy
SIL	Xerogel silica treated with ionic liquid
TEOS	Tetraethoxysilane
TG	Thermogravimetric analysis
Y_a_	Total activity recovery yield
